# Fibroblast growth factor 23 (FGF23) level is associated with ultrafiltration rate in patients on hemodialysis

**DOI:** 10.1007/s00380-020-01704-y

**Published:** 2020-09-30

**Authors:** Yoko Nishizawa, Yumi Hosoda, Ai Horimoto, Kiyotsugu Omae, Kyoko Ito, Chieko Higuchi, Hiroshi Sakura, Kosaku Nitta, Tetsuya Ogawa

**Affiliations:** 1 Department of Medicine, Tokyo Women’s Medical University Medical Center, East Tokyo 2-1-10, Nishiogu, Arakawa-ku, Tokyo 116-8567 Japan; 2 Department of Nephrology, Heisei-Hidaka Clinic, Takasaki, Gunma Japan; 3 Department of Medicine, Kidney Center, Tokyo Women’s Medical University, Shinjuku, Tokyo Japan

**Keywords:** Cardiovascular disease, Fibroblast growth factor 23, Hemodialysis, Phosphate, Ultrafiltration rate

## Abstract

Fibroblast growth factor 23 (FGF23) is a bone-derived hormone that regulates renal phosphate reabsorption and vitamin D synthesis in renal proximal tubules. High circulating FGF23 levels are associated with increased mortality in patients with chronic kidney disease and those on dialysis. Current data also suggest higher circulating levels of FGF23 are associated with cardiovascular mortality, vascular calcification, and left ventricular hypertrophy; however, evidence on the role of FGF23 in patients on dialysis is incomplete, and some of the data, especially those on cardiovascular disease (CVD), are controversial. This study aimed to evaluate factors associated with FGF23 in hemodialysis patients with or without CVD. Randomly selected 76 patients on maintenance hemodialysis at a single hemodialysis center were enrolled. After the exclusion of eight patients with extremely outlying FGF23 levels, 68 patients, including 48 males and 46 patients with a CVD history, were included in the study. The mean age was 64.4 ± 12.1 years, and the mean dialysis duration was 12.7 ± 7.1 years. Dialysis duration, time-averaged concentration of urea (TAC-urea), ultrafiltration rate (UFR), blood pressure during hemodialysis session, laboratory data, and echocardiographic parameters including interventricular septum thickness (IVST), left ventricular mass indices (LVMI), and ejection fraction were included in univariate and multivariate analyses. The median lgFGF23 levels in the overall cohort and in those with and without CVD were 2.14 (interquartile range, IQR − 0.43 to − 4.23), 2.01 (− 0.52 to 4.12), and 2.59 (0.07 to 4.32), respectively, and there was no difference between the patients with and without CVD (*p* = 0.14). The univariate analysis revealed that FGF23 was significantly associated with age (*r* =  − 0.12, *p* < 0.01), duration of hemodialysis (*r* =  − 0.11, *p* < 0.01), TAC-urea (*r* = 0.29, *p* = 0.01), UFR (*r* = 0.26, *p* = 0.04), alkaline phosphatase (ALP; *r* =  − 0.27, *p* = 0.03), corrected serum calcium (cCa; *r* = 0.32, *p* < 0.01), serum phosphate (iP, *r* = 0.57, *p* < 0.01), intact parathyroid hormone (iPTH; *r* = 0.38, *p* < 0.01), IVST (*r* = 0.30, *p* = 0.01), and LVMI (*r* = 0.26, *p* = 0.04). In multivariate regression analysis, FGF23 was significantly associated with cCa (F = 25.6, *p* < 0.01), iP (*F* = 22.5, *p* < 0.01), iPTH (*F* = 19.2, *p* < 0.01), ALP (*F* = 5.34, *p* = 0.03), and UFR (*F* = 3.94, *p* = 0.05). In addition, the univariate analysis after the categorization of patients according to CVD indicated that FGF23 was significantly associated with cCa (*r* = 0.34, *p* = 0.02), iP (*r* = 0.41, *p* < 0.01), iPTH (*r* = 0.39, *p* = 0.01), and TAC-urea (*r* = 0.45, *p* < 0.01) in patients with CVD, whereas only IVST (*r* = 0.53, *p* = 0.04) was associated with FGF23 in those without CVD. FGF23 levels in hemodialysis patients were extremely high and associated not only with mineral bone disease-related factors but also with UFR. Additionally, dialysis efficacy might be associated with lower FGF23 levels in patients with CVD.

## Introduction

Fibroblast growth factor 23 (FGF23) is a bone-derived hormone that regulates renal phosphate reabsorption and vitamin D synthesis in renal proximal tubules [1]. Importantly, FGF23 levels start increasing early in the course of chronic kidney disease (CKD) and reach extremely high levels in patients on dialysis [2–5]. Serum phosphate (iP), calcium, and intact parathyroid hormone (iPTH) are proposed to regulate FGF23 levels in uremic patients on maintenance hemodialysis [6], and current data suggest higher circulating levels of FGF23 are associated with mortality [7–11], especially cardiovascular mortality [10, 12–15] due to vascular calcification [16–19] or left ventricular hypertrophy (LVH) [20–23]. These results indicate that reducing serum FGF23 levels might improve prognosis in patients on dialysis. However, evidence on the role of FGF23 in patients on dialysis is incomplete, and some of the data, especially those on cardiovascular disease (CVD), are controversial. Therefore, we aimed to determine the factors associated with elevated serum FGF23 levels in patients on hemodialysis and to assess whether there were differences between patients with and without CVD.

## Materials and methods

### Study design and population

This cross-sectional study enrolled 76 randomly selected patients undergoing maintenance hemodialysis performed at a single hemodialysis center (Heisei Hidaka Clinic, Gunma, Japan) in April 2010. All study participants provided informed consent, and the study design was approved by the Ethical Committee on Human Research at Heisei Hidaka Clinic (No. 43). The dialysis prescriptions were hemodialysis and hemodiafiltration in 61 and 15 patients, respectively, which were administered in 4-h sessions three times weekly using a polysulfone hollow-fiber dialyzer (APS or ABH; Asahi Kasei Medical, Tokyo, Japan). Blood and dialysate flows were 120–250 mL/min and 500 mL/min, respectively, with a constant ultrafiltration rate (UFR). The dialysate bath comprised 140 mmol/L sodium, 2.0 mmol/L potassium, 2.5 mmol/L calcium, 1.0 mmol/L magnesium, 8.0 mmol/L acetate, 25.0 mmol/L bicarbonate, and 150 mg/dL glucose (Kindaly 3D; Fuso, Osaka, Japan). Blood samples were collected at the start of dialysis at the end of the longest interdialytic interval, and echocardiographic parameters were measured at the end of a regular 4-h hemodialysis session on the last session of the week.

### Clinical and laboratory measurements

All blood samples were collected immediately before the hemodialysis session, as previously described, quickly mixed with 5 mg edetic acid, and centrifuged at 3000 rpm for 5 min to separate plasma. Plasma samples were stored at − 20 °C until analysis. Serum intact FGF23 concentrations were measured with a two-step FGF23 enzyme immunoassay (ELISA) kit (Kainos Laboratories Inc., Tokyo, Japan). Additionally, associated laboratory data, including levels of C-reactive protein, alkaline phosphatase (ALP), corrected serum calcium (cCa), iP, and iPTH, were measured by routine laboratory tests. Clinical data, including age, sex, dialysis duration, time-averaged concentration of urea (TAC-urea), UFR, blood pressure during hemodialysis session, and echocardiographic parameters, including interventricular septum thickness (IVST), left ventricular mass indices (LVMI), and ejection fraction, were also collected.

### Statistical analysis

Based on the measurement of intact FGF23 levels, patients who were extreme outliers (*n* = 8) were excluded, and 68 patients were included in the final analysis. Among these patients, 46 patients had a history of CVD. All statistical analyses were performed using JMP Pro version 12 for Mac (SAS Institute Japan, Tokyo, Japan). Data were reported as means ± standard deviation for normally distributed data and numbers with percentages for nominal data. A two-sided *p* value of < 0.05 was considered to indicate statistical significance. Univariable and multivariable linear regression analyses were performed to determine factors associated with natural log-transformed (Ln) serum FGF23 levels in patients on hemodialysis. Additional analyses were performed to determine factors associated with Ln serum FGF23 based on the presence of CVD.

## Results

**Fig. 1:**
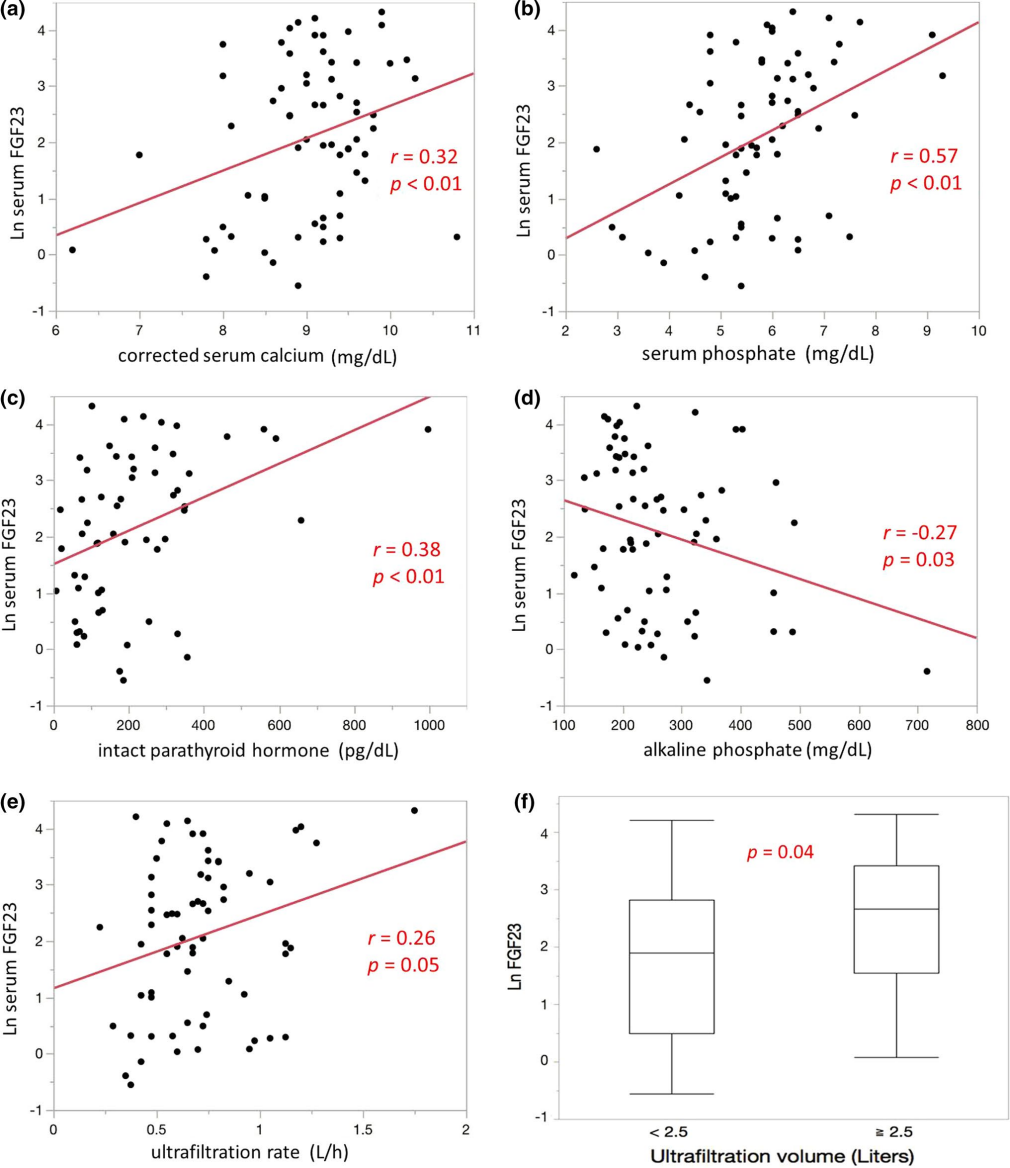
Relationship between serum FGF23 and associated factors by multiple regression analysis. In multiple regression analysis, corrected serum calcium (**a**), serum phosphate (**b**), intact parathyroid hormone (**c**), alkaline phosphate (**d**), and ultrafiltration rate (**e**) were associated with serum FGF23. Higher ultrafiltration volume is associated with higher serum FGF23 level by subgroup analysis (*p* = 0.04) (**f**). *FGF23* fibroblast growth factor 23

**Fig. 2:**
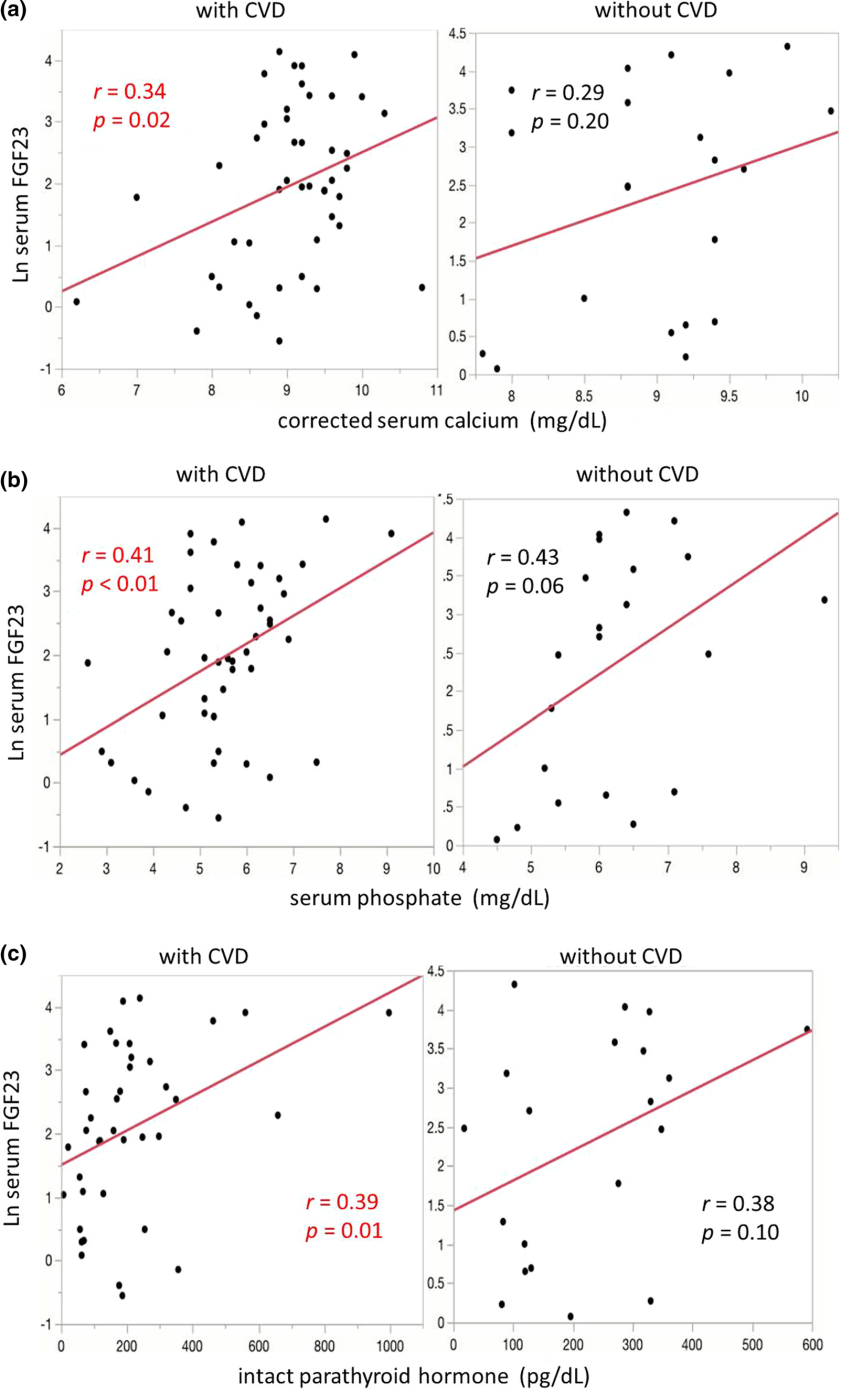

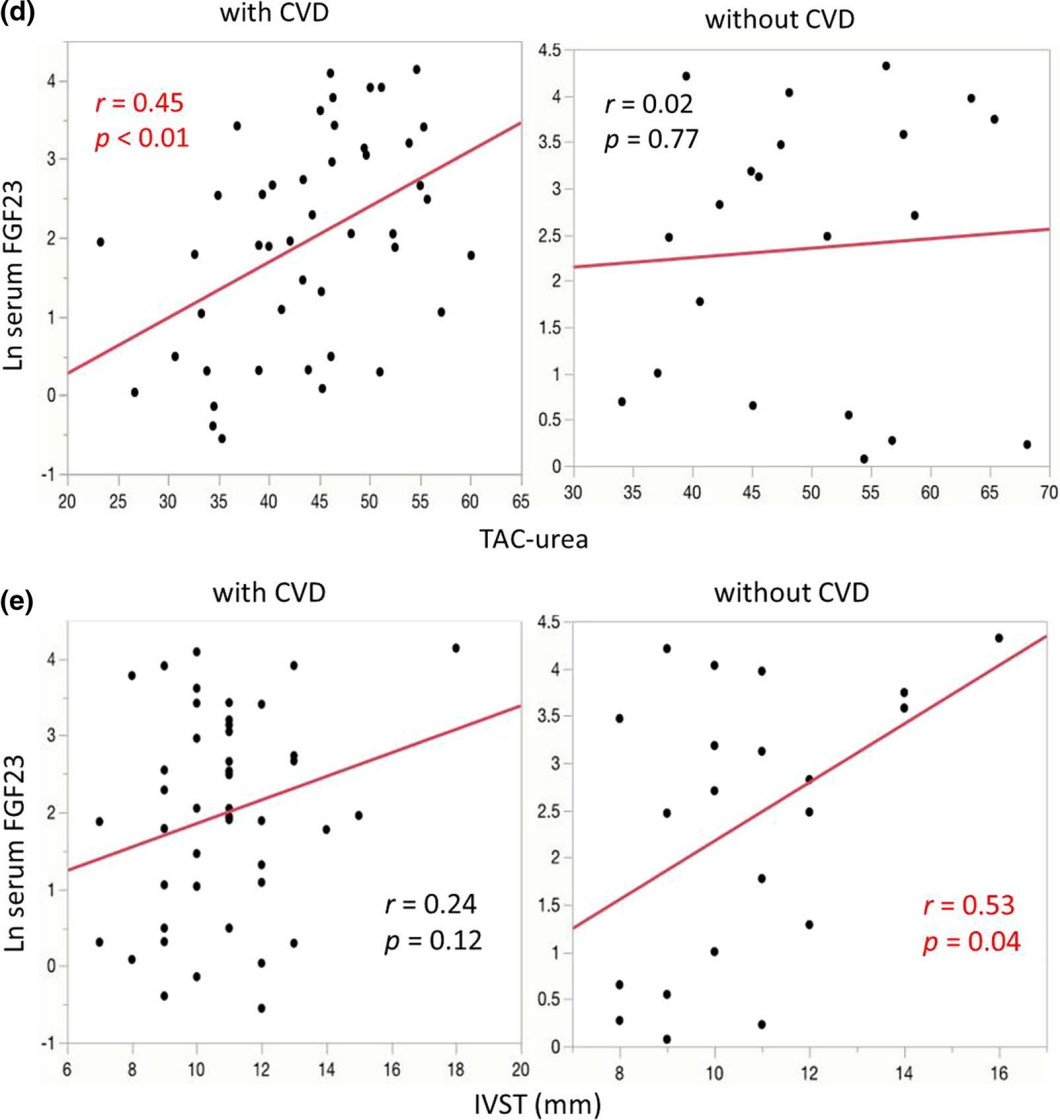
Relationship between serum FGF23 and basic patients’ characteristics with or without CVD. In patients with CVD (left), corrected serum calcium (**a**), serum phosphate (**b**), intact parathyroid hormone (**c**), and TAC-urea (**d**) were associated with FGF23, whereas only IVST (**e**) was associated with serum FGF23 in patients without CVD (right). *CVD* cardiovascular disease; *FGF23* fibroblast growth factor 23, *IVST* interventricular septum thicknesses, *TAC-urea* time-averaged concentration of urea

The baseline patient characteristics are presented in Table 1. The median levels of FGF23 in the overall cohort and those with and without CVD were 2.14 (interquartile range, IQR − 0.43 to − 4.23), 2.01 (− 0.52 to 4.12), and 2.59 (0.07 to 4.32), respectively, with no significant difference observed between the patients with and without CVD (*p* = 0.14). We analyzed the relationship between serum FGF23 and the clinical parameters. In univariate analysis, age (*r* =  − 0.12, *p* < 0.01), duration of hemodialysis (*r* =  − 0.11, *p* < 0.01), TAC-urea (*r* = 0.29, *p* = 0.01), UFR (*r* = 0.26, *p* = 0.04), alkaline phosphatase (ALP; *r* =  − 0.27, *p* = 0.03), corrected serum calcium (cCa; *r* = 0.32, *p* < 0.01), serum phosphate (iP, *r* = 0.57, *p* < 0.01), intact parathyroid hormone (iPTH; *r* = 0.38, *p* < 0.01), IVST (*r* = 0.30, *p* = 0.01), and LVMI (*r* = 0.26, *p* = 0.04) were associated with FGF23, as shown in Table 2. Multiple regression analysis to determine the association of these factors with serum FGF23 revealed that cCa (*F* = 25.6, *p* < 0.01), iP (*F* = 22.5, *p* < 0.01), iPTH (*F* = 19.2, *p* < 0.01), ALP (*F* = 5.34, *p* = 0.03), and UFR (*F* = 3.94, *p* = 0.05) were associated with FGF23 (Fig. 1). We divided the study group into two subgroups by median ultrafiltration volume, and it was associated with serum FGF23 level (*p* = 0.04). In addition, univariate analysis performed after the classification of patients according to the presence of CVD revealed that cCa (*r* = 0.34, *p* = 0.02), iP (*r* = 0.41, *p* < 0.01), iPTH (*r* = 0.39, *p* = 0.01), and TAC-urea (*r* = 0.45, *p* < 0.01) were associated with serum FGF23 in patients with CVD, whereas only IVST (*r* = 0.53, *p* = 0.04) was associated with serum FGF23 in patients without CVD (Table 3; Fig. 2).

**Table 1:** Baseline patient characteristics of study population

	Overall (*N* = 68) Median (IQR)	with CVD (*N* = 46) Median (IQR)	without CVD (*N* = 22) Median (IQR)	*p* value
Age (years)	65 (36–83)	66 (38–84)	65 (34–83)	NS
Male: female (*N*)	48: 20	34: 12	14: 8	NS
Duration of hemodialysis (years)	11.2 (4.97–30.6)	11.1 (4.9–31.6)	12.4 (5.0–27.2)	NS
Primary disease of hemodialysis				
Diabetes mellitus	21 (30.8%)	15 (32.6%)	6 (27.3%)	
Chronic glomerulonephritis	32 (47.1%)	22 (47.8%)	10 (45.5%)	
Nephrosclerosis	6 (8.8%)	5 (10.8%)	1 (4.5%)	
Medications				
Angiotensin receptor antagonist	35 (51.5%)	26 (56.5%)	8 (36.3%)	
Calcium antagonist	25 (36.7%)	16 (34.7%)	9 (40.8%)	
Beta-blocker	12 (17.6%)	10 (21.7%)	2 (9.1%)	
Statin	4 (5.8%)	3 (6.5%)	1 (4.5%)	
Uric acid synthesis inhibitor	3 (13.6%)	0	3 (13.6%)	
Potassium absorptive agent	15 (22.1%)	10 (21.7%)	5 (22.7%)	
Vitamin D	20 (29.4%)	13 (28.2%)	7 (31.8%)	
Calcium carbonate	61 (89.7%)	41 (89.1%)	20 (90.9%)	
Lanthanum	12 (17.6%)	6 (13.0%)	6 (27.3%)	
Sevelamer	26 (38.2%)	18 (39.1%)	8 (36.3%)	
Cinacalcet	21 (30.8%)	12 (26.1%)	9 (40.9%)	
Systolic blood pressure (mmHg)	146 (99–185)	144 (77–188)	146 (125–184)	NS
Ultrafiltration rate (l/h)	0.675 (0.26–1.44)	0.67 (0.23–1.14)	0.71 (0.4–1.75)	NS
lgFGF23	2.14 (–0.43 to 4.23)	2.01 (–0.52 to 4.12)	2.59 (0.07 to 4.32)	NS
TAC-urea (mg/dl)	45.5 (25.6–66.3)	45.1 (23.8–59.6)	48.2 (34.1–68.2)	0.01
C-reactive protein (mg/dl)	0.12 (0.02–3.70)	0.12 (0.02–4.28)	0.11 (0.02–1.02)	NS
Alkaline phosphate (mg/dl)	236 (130–552)	236 (120–676)	236 (156–456)	NS
Corrected serum calcium (mg/dl)	9.2 (6.8–10.1)	9.2 (6.3–10.7)	9.1 (7.8–10.2)	NS
Serum phosphate (mg/dl)	5.8 (2.8–9.2)	5.4 (2.6–8.8)	6.0 (4.5–9.3)	0.03
Intact parathyroid hormone (pg/dl)	179 (13–828)	176 (7–997)	233 (18–592)	NS
IVST (mm)	11 (7–17)	11 (7–17)	10 (8–16)	NS
LVMI (g/m^2^)	106 (65–193)	111 (60–212)	98 (68–178)	NS
Ejection fraction (%)	65 (31–84)	64 (26–86)	66 (48–81)	NS

*CVD* cardiovascular disease, *FGF23* fibroblast growth factor 23, *IQR* interquartile range, *IVST* interventricular septum thicknesses, *LVMI* left ventricular mass indexes, *TAC-urea* time-averaged concentration of urea

**Table 2:** Relationship between serum FGF23 and basic characteristics of study population

	Univariate analysis, *r*	*p* value	Multiple regression analysis, *F*	*p* value
Age (years)	– 0.12	< 0.01		
Male: female (*N*)	– 0.03	0.41		
Duration of hemodialysis (years)	– 0.11	< 0.01		
TAC-urea (mg/dl)	0.29	0.01	0.08	0.78
Ultrafiltration rate (l/h)	0.26	0.04	3.94	0.05
Blood pressure (mmHg)	0.17	0.17		
C-reactive protein (mg/dl)	– 0.04	0.72		
Alkaline phosphate (mg/dl)	– 0.27	0.03	5.34	0.03
Corrected serum calcium (mg/dl)	0.32	< 0.01	25.6	< 0.001
Serum phosphate (mg/dl)	0.57	< 0.01	22.5	< 0.001
Intact parathyroid hormone (pg/dl)	0.38	< 0.01	19.2	< 0.001
IVST (mm)	0.30	0.01	0.04	0.83
LVMI (g/m^2^)	0.26	0.04	1.13	0.29
Ejection fraction (%)	0.05	0.86		

*CVD* cardiovascular disease, *IVST* interventricular septum thicknesses, *LVMI* left ventricular mass indexes, *TAC-urea* time-averaged concentration of urea

**Table 3:** Relationship between serum FGF23 and basic patients’ characteristics with or without CVD

	With CVD		Without CVD	
	*R*	*p* value	*r*	*p* value
Age (years)	0.01	0.94	– 0.24	0.27
Duration of hemodialysis (years)	– 0.16	0.29	0.02	0.91
TAC-urea	0.45	< 0.01	0.02	0.77
Ultrafiltration rate (l/h)	0.22	0.14	0.28	0.24
Blood pressure (mmHg)	0.11	0.47	0.28	0.21
C-reactive protein (mg/dl)	– 0.06	0.71	0.26	0.70
Alkaline phosphate (mg/dl)	– 0.25	0.10	– 0.33	0.13
Corrected serum calcium (mg/dl)	0.34	0.02	0.29	0.20
Serum phosphate (mg/dl)	0.41	< 0.01	0.43	0.06
Intact parathyroid hormone (pg/dl)	0.39	0.01	0.38	0.10
IVST (mm)	0.24	0.12	0.53	0.04
LVMI (g/m^2^)	0.21	0.16	0.39	0.08

*CVD* cardiovascular disease, *IVST* interventricular septum thicknesses, *LVMI* left ventricular mass indexes, *TAC-urea* time-averaged concentration of urea

## Discussion

Serum levels of FGF23, a phosphaturic hormone that suppresses 1,25(OH)_2_-vitamin D_3_ production in kidneys [24], increase early in the course of CKD and reach levels that are several hundred times the normal range in patients with advanced CKD and end-stage renal disease (ESRD) [2]. Additionally, high levels of FGF23 have recently emerged as one of the strongest predictors of adverse outcomes in patients with CKD and ESRD [25]. The mean FGF23 level of the overall cohort (17,039 ± 19,178 pg/mL) was significantly higher than the reported normal range. Conversely, the mean serum levels of FGF23 did not differ significantly between patients with and without CVD. Despite an ever-expanding pool of observational data suggesting the potential contribution of FGF23 to CVD and mortality, current reviews and meta-analyses [26–28] suggest that the current evidence does not support a causal relationship between FGF23 and adverse events in patients with ESRD or in those with normal kidney function. The results of the current study also failed to find an association between serum FGF23 level and CVD.

Our univariate analysis also revealed that age, duration of hemodialysis, TAC-urea, UFR, cCa, iP, ALP, iPTH, IVST, and LVMI were associated with serum FGF23 in patients on maintenance hemodialysis. We also found that cCa, iP, iPTH, ALP, and UFR were associated with serum FGF23 in multiple regression analysis. As a phosphaturic hormone, FGF23 is stimulated by decreased renal phosphate excretion and subsequent hyperphosphatemia due to a declining glomerular filtration rate in patients with CKD [25]; therefore, it is reasonable that these markers related to iP homeostasis (cCa, iP, ALP, and iPTH) were associated with serum FGF23. IVST and LVMI were also associated with serum FGF23; this finding might reflect LVH in these patients on dialysis. Several studies have reported the association between FGF23 and LVH [20–23]. Intramyocardial or intravenous injection of FGF23 in wild-type mice was shown to lead to LVH [29], whereas FGF23-induced LVH in patients with CKD might be independent of blood pressure, indicating differences in the cause of LVH between patients with cardiorenal syndrome and those without primary kidney damage [30]. Additionally, LVH was reported to contribute independently to elevations in FGF23 levels [31]. In the setting of experimental myocardial infarction in mice, Andhrukova et al. [32] found that circulating FGF23 levels were increased with a concomitant reduction in the level of 1,25(OH)_2_-vitamin D_3_ and that myocardial FGF23 mRNA and protein levels were increased, suggesting that the observed increase in circulating FGF23 levels after myocardial infarction was at least partially derived from the myocardium itself [31]. In fact, a recent review on FGF23 [33] has concluded hypothesis on mono-directional effect that external factors stimulate bone FGF23 release, which in turn induces myocardial damage is challenging. Furthermore, Grabner et al. [34] revealed that specific FGF receptor 4 (FGFR4) inhibition attenuated the established LVH in the rat 5/6 nephrectomy model of CKD and demonstrated that aging mice lacking FGFR4 were protected from LVH. These lines of evidence suggest that FGF23-induced cardiac hypertrophy is reversible in vitro and in vivo*,* following the removal of hypertrophic stimulus, and indicate that pharmacological interference with cardiac FGF23/FGFR4 signaling might provide protection from CKD- and age-related LVH [35]. Indeed, Leifheit-Nestler et al*.* [36] reported that vitamin D treatment attenuated cardiac FGF23/FGFR4 signaling and hypertrophy in uremic rats. However, a reduction of FGF23 as a therapeutic option in kidney or cardiac disease is currently not appealing since the potential chronic effects of FGF23 inhibition are unpredictable in conditions with reactive, secondary FGF23 elevation [33].

The current study also revealed a relationship between serum FGF23 level and hemodialysis efficacy. The results of the limited number of studies to date have evaluated that the relationship between dialysis efficacy and FGF23 supports the current study findings. Zaritsky et al. [37] reported that short daily hemodialysis sessions were associated with lower plasma FGF23 levels compared with conventional hemodialysis, whereas Hacıhamdioglu et al. [38] reported that FGF23 levels were associated with effective dialysis in children on peritoneal dialysis. Together with our findings, these data suggest that more effective dialysis might lower FGF23. Additionally, we have revealed a relationship between high UFR levels and high FGF23 levels. Recent studies suggest a role for FGF23 in volume regulation. Andrukhova et al. [39] showed that FGF23 increased the membrane abundance and phosphorylation of Na^+^–Cl^−^ cotransporter and induced Na^+^ uptake in distal renal tubules in vivo and in vitro using recombinant FGF23-administered mice; the authors concluded that FGF23 was a key regulator of renal Na^+^ reabsorption and plasma volume. One study [40] found that elevated FGF23 correlated with hypervolemia in patients on hemodialysis, although another study [41] reported that FGF23, albeit correlating with volume status in patients on hemodialysis, was not reduced by the hemodialysis.

We performed additional univariate analysis to compare parameters associated with FGF23 based on the presence of CVD and found that cCa, iP, iPTH, and TAC-urea were associated with FGF23 in patients with CVD but not in those without CVD. One potential reason for these observed differences might be the variations in baseline patient characteristics between the two groups. Specifically, TAC-urea levels were significantly lower, and serum iP levels were significantly higher in patients with CVD in the present study. Vascular calcification in patients on dialysis was reported to be associated with FGF23 [16–19], whereas FGF23 was shown to decline after 3 h of hemodialysis [42]; these findings suggest that lowering circulating FGF23 levels by increasing dialysis efficacy might prevent or lower CVD events in patients on dialysis. Conversely, several studies [43–45] reported that there was no evident association between vascular calcification and FGF23, and there is currently no direct evidence supporting a causal relationship between FGF23 and cardiovascular events [26–28]. Therefore, the current study findings do not conclusively show that increasing dialysis efficacy and restricting the control of CKD-mineral bone disease might reduce circulating FGF23 levels in patients with CVD. As studies on dialysis patients are few today, further investigation is warranted.

## Conclusions

FGF23 levels, which were extremely higher than the normal range in patients on hemodialysis, were associated not only with mineral bone disease-associated factors but also with UFR. Additionally, increased dialysis efficacy might be associated with lower FGF23 in patients with CVD.
